# Draft Genome Sequence of *Micrococcus luteus* (Schroeter) Cohn (ATCC 12698)

**DOI:** 10.1128/genomeA.00576-17

**Published:** 2017-07-06

**Authors:** Catherine Putonti, Evan Cudone, Laurynas Kalesinskas, Kathleen C. Engelbrecht, David W. Koenig, Alan J. Wolfe

**Affiliations:** a Bioinformatics Program, Loyola University Chicago, Chicago, Illinois, USA; b Department of Biology, Loyola University Chicago, Chicago, Illinois, USA; c Department of Computer Science, Loyola University Chicago, Chicago, Illinois, USA; d Department of Microbiology and Immunology, Stritch School of Medicine, Health Sciences Division, Loyola University Chicago, Maywood, Illinois, USA; e Corporate Research and Engineering, Kimberly-Clark Corporation, Neenah, Wisconsin, USA

## Abstract

The actinobacterium *Micrococcus luteus* can be found in a wide variety of habitats. Here, we report the 2,411,958-bp draft genome sequence of the type strain *M. leuteus* (Schroeter) Cohn (ATCC 12698). Characteristic of this taxa, the genome sequence has a high G+C content, 73.14%.

## GENOME ANNOUNCEMENT

Strains of the actinobacterium *Micrococcus luteus* have been isolated from a wide variety of habitats, including soil (1) and the microbiota of mammalian skin (2). Genomic sequences derived from these different sources provide insight into the diversity of this species (2–4). Here, we report the genome sequence and annotation of the type strain *Micrococcus luteus* (Schroeter) Cohn (ATCC 12698).

The culture isolate was purchased from ATCC and grown on 5% sheep blood agar (BD BBL prepared plated media) under 5% CO_2_ for 48 h at 35°C. To extract genomic DNA, cells were resuspended in 0.5 mL of DNA extraction buffer (20 mM Tris-Cl, 2 mM EDTA, 1.2% Triton X-100, pH 8), followed by the addition of 50 µL of lysozyme (20 mg/mL), 30 µL of mutanolysin, and 5 µL of RNase (10 mg/mL). After a 1-h incubation at 37°C, 80 μL of 10% SDS and 20 µL of proteinase K were added. This mixture was then incubated for 2 h at 55°C. Then, 210 μL of 6M NaCl and 700 μL of phenol-chloroform were added. After a 30-min incubation with rotation, the solutions were centrifuged at 13,500 rpm for 10 min, and the aqueous phase was extracted. An equivalent volume of isopropanol was added and incubated for 10 min prior to centrifugation at 13,500 rpm for 10 min. The supernatant was decanted, and the DNA pellet was precipitated using 600 μL of 70% ethanol. Following ethanol evaporation, the DNA pellet was resuspended in Tris-EDTA (TE) and stored at −20°C.

Genomic DNA was diluted in water to a concentration of 0.2 ng/µL, as measured by a fluorometric-based method (Life Technologies, Inc.). Library preparation of 1 ng (5 μL) of isolated DNA was performed using the Nextera XT DNA library preparation kit. The library was sequenced on the MiSeq sequencer (Illumina) using the MiSeq version 2 reagent kit (500 cycles), and 1,645,841 paired-end reads were produced. Reads were first processed, removing adapter sequences and phiX contaminants, using BBDuk from the BBMap package (http://sourceforge.net/projects/bbmap). Trimmed reads were assembled using SPAdes version 3.5 (5) and scaffolded using SSPACE (6). This resulted in 126 contigs at an average coverage of 268.7×. Contigs varied in length from 1,181 bp to 149,560 bp (*N*_50 _= 31,560 bp). The total genome size was 2,411,958 bp with an observed G+C content of 73.14%. Annotations were produced using the software tool Peasant (7). Five rRNAs, 48 tRNAs, and 2,317 protein-coding sequences were detected. No regularly interspaced short palindromic repeat sequences (CRISPRs) were found within the *M. luteus* strain Cohn (ATCC 12698) genomic sequence (8); to date, the CRISPR/Cas system has not been identified in this taxon (9). The assembly for the ATCC 12698 strain and the representative genome for the strain *M. luteus* NCTC 2665 (GenBank no. NC_012803.1) were compared using Mauve (10), revealing polymorphisms as well as regions unique to each strain.

### Accession number(s).

The draft whole-genome project for *M. luteus* strain Cohn (ATCC 12698) has been deposited at DDBJ/EMBL/GenBank under the accession number NAQZ00000000. Raw sequence reads were deposited at DDBJ/EMBL/GenBank under the accession number SRR5364946.
